# Barriers From Third-Party Payers to Biologic Use in Pediatric Inflammatory Bowel Disease

**DOI:** 10.1097/PG9.0000000000000215

**Published:** 2022-06-21

**Authors:** Chelsea A. Lepus, Jeffrey S. Hyams

**Affiliations:** From the Division of Pediatric Gastroenterology, Hepatology, and Nutrition, Connecticut Children’s Medical Center, Hartford, CT.

**Keywords:** biologic therapy, inflammatory bowel disease

## Abstract

Biologic agents are now standard of care in the treatment of inflammatory bowel disease (IBD). The ability to use biologics in clinical practice is in part dictated by insurance company policies. There is a long delay between adult and pediatric approval of biologic agents, and these therapies are often denied by third-party payers for use in pediatric IBD patients. This study prospectively identified pediatric patients with IBD who were started on a biologic medication at our institution, and third-party payer decisions were recorded. There were no denials in patients with Medicaid, but private payers frequently interfered with use of biologic agents. Reasons for denial are generally for use of a specific off-label agent or dosing of an approved agent. These denials lead to delayed treatment, nonmedically sound changes in therapy, and increased administrative burden on providers.

**What Is Known**

- Inflammatory bowel disease (IBD) is a condition of chronic intestinal inflammation, and the treatment goal is to achieve remission by demonstrating complete mucosal healing.
- Biologic agents are widely used in the treatment of IBD, although there are long delays in pediatric approval of biologic agents from the time of approval in adults.

**What Is New**

- Third-party private payers frequently interfere in the use of biologic agents in pediatric patients with IBD.
- Reasons for denial are generally for use of a specific off-label agent or dosing of an approved agent.

## INTRODUCTION

Inflammatory bowel disease (IBD) is a condition of chronic intestinal inflammation, and the treatment goal is to achieve remission by achieving complete mucosal healing.^1–3^ Biologic agents are now widely used to attain this goal, and while there are a growing number of therapies approved for use in adults, there is a long delay between adult and pediatric approvals, which leads to years of off-label use of these medications in pediatric patients. Therapeutic drug monitoring (TDM) is now standard of care for optimizing biologic therapy with adult studies demonstrating improved therapeutic outcomes with proactive TDM.^4,5^ TDM often leads to dosing above that described on a drug label. The concept of dose escalation to achieve therapeutic goals was not operative at the time of drug registration and third-party payers often deem dosing changes guided by TDM as “experimental.” We describe a prospective accounting of barriers from third-party payers to the use of biologic agents in pediatric patients with IBD at our institution.

## METHODS

Prior authorization requests for pediatric and adolescent patients with IBD who were started on a biologic medication at Connecticut Children’s from January 1, 2021, to September 1, 2021, were prospectively identified and reviewed as they progressed. Demographic and clinical data including specific agent and dosing requests were recorded along with third-party payer decisions. Continuous variables were summarized in means ± SDs and categorical variables in proportions or percentages. This study was approved by Connecticut Children’s Institutional Review Board.

## RESULTS

One-hundred eight IBD patients were started on a biologic medication during the study period, mean ± SD age 14 years (±5 years), 66% male, 59% Crohn’s disease, and 41% ulcerative colitis/IBD-unclassified. Seventy-four percent had private insurance, 26% Medicaid. Biologics included infliximab (58 patients), infliximab-dyyb (7 patients), vedolizumab (25 patients), ustekinumab (17 patients), and adalimumab (1 patient). Thirty-one patients (29%) had previously been on another biologic agent; 12% infliximab (7 patients), 14% infliximab-dyyb (1 patient), 28% vedolizumab (7 patients), 88% ustekinumab (15 patients), and 100% adalimumab (1 patient).

**FIGURE 1.**
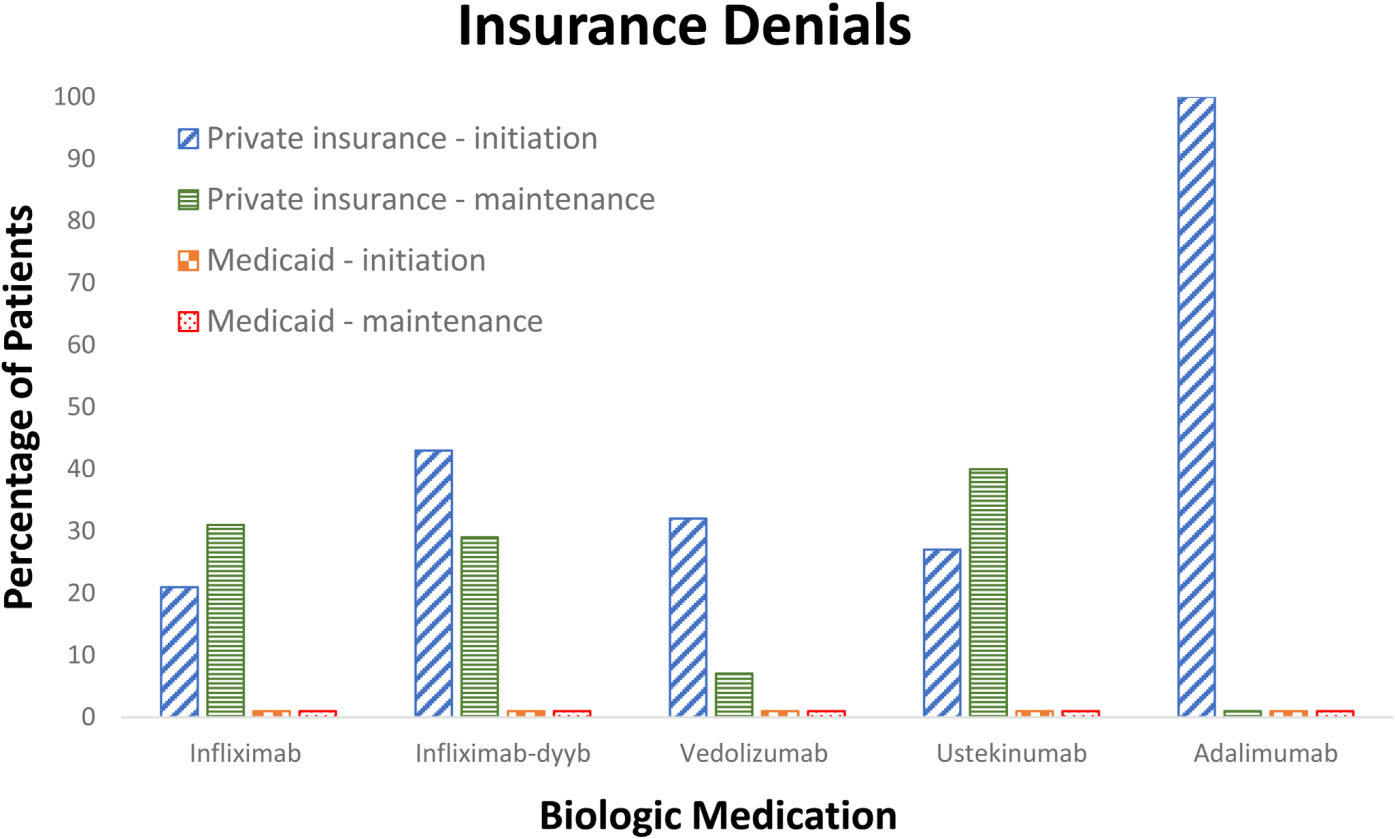
Percentage of patients with insurance denials by biologic medication for both therapy plan initiation (induction) and maintenance adjustments, comparing private insurance to Medicaid.

There were no denials in patients with Medicaid. For infliximab, there were 8 denials after therapy plan initiation (TPI), which required either dose change (6 patients) or change to biosimilar (2 patients). The denials that required dose change were all due to requested doses over 5 mg/kg (ranging from 6.5 to 10 mg/kg) and were approved when dose was adjusted to 5 mg/kg. After infliximab induction, a change in either dose or interval for maintenance therapy was made in 41 patients based on suboptimal clinical efficacy (9 patients) or TDM obtained prior to the third dose showing subtherapeutic levels (32 patients). Of these requested changes, there were 11 denials requiring change in dose or interval from treating physician preference (2 patients), change to biosimilar (5 patients), or required a peer to peer (P2P) for approval (4 patients). For infliximab-dyyb, there were 3 denials after TPI, requiring dose change (1 patient) or P2P (2 patients). The denial that required dose change was due to requested dose of 7.4 mg/kg and was approved when the dose was adjusted to 5 mg/kg. Of the 2 patients requiring P2P, one was approved for 10 mg/kg dosing and the other was approved after the requested dose was adjusted from 8.7 mg/kg to 5 mg/kg. After infliximab-dyyb induction, a change in either dose or interval for maintenance therapy was made in 2 patients based on suboptimal clinical efficacy (1 patient) or subtherapeutic level (1 patient), which were both initially denied. The latter was approved after P2P, but the symptomatic patient was hospitalized and underwent change in therapy. For vedolizumab, there were 6 denials after TPI. All underwent P2P and/or appeal, with 3 approvals and 3 denials. The 3 denials were in patients 12–14 years of age and required switch to infliximab. For ustekinumab, there were 4 denials after TPI (all were patients ages 16–17 who had failed prior biologic therapy), all approved after P2P, with 6 denials for subsequent subcutaneous dosing frequency changes all of which were approved after P2P. The 1 patient started on adalimumab initially had a denial that was approved upon appeal. Figure 1 depicts a summary.

The average time between TPI and biologic initiation (first infusion) was 9.7 days (±3.7 days) for patients with Medicaid, 11.3 days (±5.2 days) for patients with private insurance who had approvals, and 18.8 days (±7.6 days) for patients with private insurance who initially had denials.

## DISCUSSION

Barriers from third-party payers to the use of biologic agents in pediatric patients with IBD are common and denials are generally due to use of a specific off-label agent or dosing of an approved agent. These denials often lead to delayed treatment and nonmedically sound changes in therapy, which can potentially lead to complications and increased IBD-related healthcare utilization.

Long delays in pediatric approval of biologic agents from the time of approval in adults lead to off-label use in children. Adult and then pediatric approval for infliximab was 24 and 16 years ago, respectively.^6^ Since that time, the concept of TDM to ensure adequate drug exposure, prevention of anti-drug antibody development, and eventual bowel healing ^7,8^ has become part of standard practice, especially for anti-tumor necrosis factor therapy. Proactive TDM with drug titration to a target trough concentration is associated with better therapeutic outcomes when compared with standard of care or reactive TDM.^4,5^ In our cohort of patients started on infliximab, there was a requested change in dose or interval for maintenance therapy in 32 patients due to subtherapeutic levels, many of which were denied. The concept of TDM was not part of the original drug label for infliximab and is used by third-party payers to deny changes related to drug levels. Despite the growing body of literature supporting use of TDM, third-party private payers customarily deny adjustments unless the patient is symptomatic, a situation that clinicians try to avoid.

Vedolizumab and ustekinumab were approved in 2014 and 2016 for adults with IBD and have not yet been approved for pediatric use but are commonly used off-label.^9^ Vedolizumab is a gut-selective antibody to α_4_β_7_ integrin and has been shown to have a favorable safety profile over an extended treatment period with low incidence rates of serious infections, infusion-related reactions, and malignancies.^10^ In our cohort, there were 3 patients ages 12–14 with newly diagnosed ulcerative colitis pancolitis or Crohn’s colitis in which physicians deemed vedolizumab to be the most clinically appropriate therapy. Despite this, all 3 patients were required to start infliximab due to denial of vedolizumab initiation. Although these medications may be more effective in certain clinical scenarios and safer than older medications, third-party payers often consider them “experimental” and deny their use.

In our study, the average time from TPI to biologic initiation was 9.1 days longer in patients with private insurance who initially had denials compared with those with Medicaid (18.8 versus 9.7 days, respectively). Another recent study found that prior authorization and complicated authorizations requiring appeal, step therapy, or P2P were associated with 10.2- and 24.6-day increases in biologic initiation time, respectively.^11^ In that same study, prior authorizations were associated with a 12.9% increased likelihood of IBD-related healthcare utilization within 180 days of biologic recommendation and a 14.1% increased likelihood of corticosteroid dependence at 90 days.

There are many administrative obstacles to overcome in optimizing the use of biologic medications in children with IBD. Clinicians are often denied the use of evidence-based practice to optimize therapy and need to adhere to drug label guidelines that are decades outdated, which can contribute to provider frustration and burnout. It is imperative that professional societies engage third-party payers to create less burdensome pathways to ensure that pediatric patients can receive the benefit of advancing science in the use of these medications.

## ACKNOWLEDGMENTS

C.A.L. contributed to the conception and design of the study, collection and interpretation of data, and drafted and critically revised the article. J.S.H. contributed to the conception and design of the study, interpretation of the data, and critically revised the article. C.A.L. is accepting full responsibility for the conduct of the study (guarantor of the article). All authors agree to be fully accountable for ensuring the integrity and accuracy of the work and read and approved the final article.
